# Translational evidence for the involvement of the endocannabinoid system in stress-related psychiatric illnesses

**DOI:** 10.1186/2045-5380-3-19

**Published:** 2013-10-22

**Authors:** Matthew N Hill, Sachin Patel

**Affiliations:** 1 Hotchkiss Brain Institute, Departments of Cell Biology & Anatomy and Psychiatry, University of Calgary, 3330 Hospital Drive NW, Calgary AB T2N4N1, Canada; 2 Departments of Psychiatry, and Molecular Physiology and Biophysics, Vanderbilt University School of Medicine, 724B Robinson Research Building, Nashville, TN, USA

**Keywords:** Endocannabinoid, Anandamide, 2-AG, Stress, Anxiety, Depression, PTSD

## Abstract

Accumulating evidence over the past decade has highlighted an important role of the endocannabinoid (eCB) system in the regulation of stress and emotional behavior across divergent species, from rodents to humans. The general findings from this work indicate that the eCB system plays an important role in gating and buffering the stress response, dampening anxiety and regulating mood. Work in rodents has allowed researchers to determine the neural mechanisms mediating this relationship while work in human populations has demonstrated the possible importance of this system in stress-related psychiatric diseases, such as post-traumatic stress disorder, generalized anxiety and major depression. These stress-protective effects of eCB signaling appear to be primarily mediated by their actions within corticolimbic structures, particularly the amygdala and the prefrontal cortex. The aim of this review is to provide an up-to-date discussion of the current level of knowledge in this field, as well as address the current gaps in knowledge and specific areas of research that require attention.

## Review

For well over half a century investigation of the biological mechanisms subserving emotional behavior and affective disorders has focused heavily on the role of brain monoaminergic signaling. Indeed, dopamine, norepinephrine and serotonin have all been implicated in the pathophysiology of anxiety disorders and major depressive disorder, and the vast majority of current pharmacotherapies for affective illnesses target monoaminergic systems. However, more recent studies have begun to highlight alternative neurochemical systems in the regulation of mood and anxiety including neuropeptides, cytokines and bioactive lipids.

**Figure 1:**
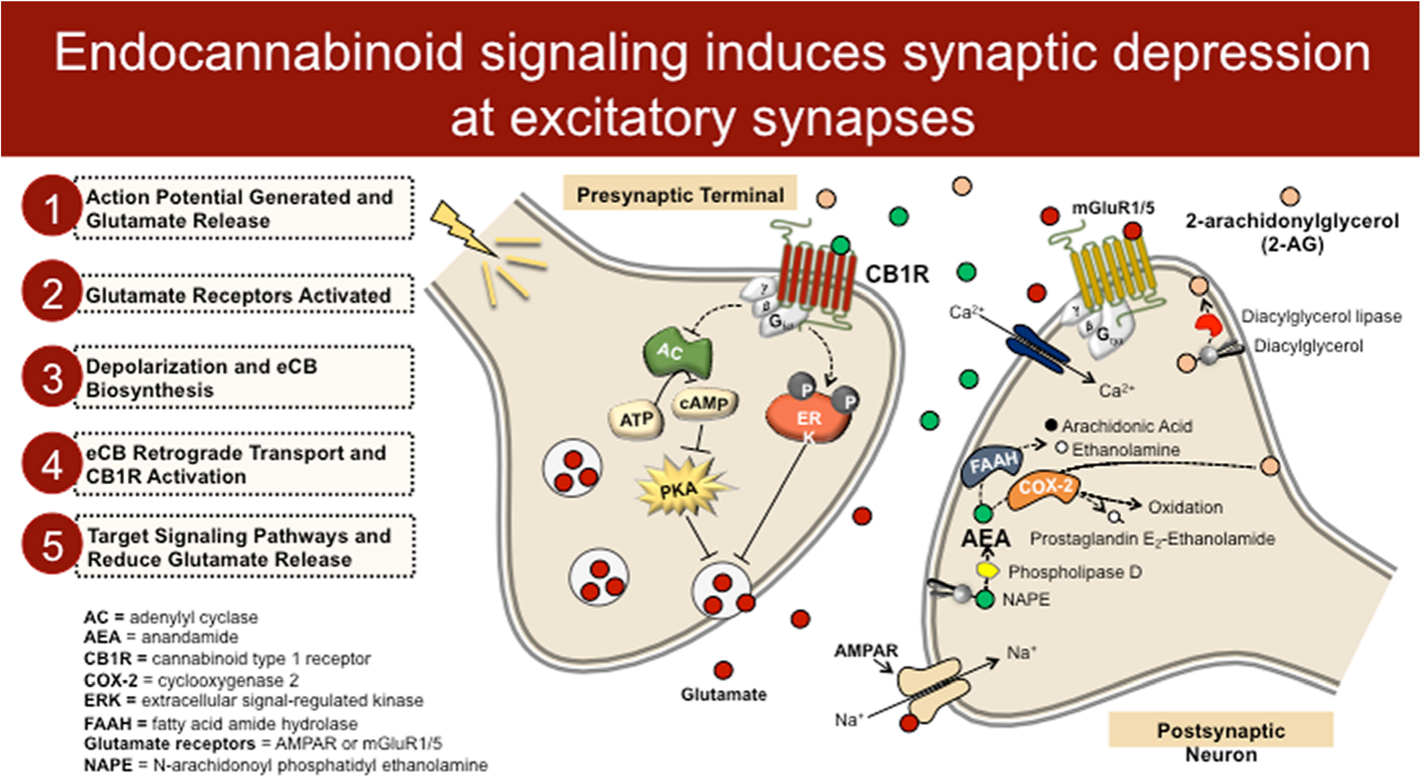
**Molecular architecture of endocannabinoid signaling at an excitatory synapse in the brain.** 2-AG, 2-arachidonoylglycerol; AEA, anandamide; CB1, cannabinoid type 1; FAAH, fatty-acid amide hydrolase; MAGL, monoacylglycerol lipase.

Endogenous cannabinoids (eCBs) are one class of bioactive lipids produced in the brain and periphery that exert biological actions via activation of cannabinoid type 1 (CB1) and 2 (CB2) receptors. CB1 receptors are found primarily in the brain on axon terminals of most neurochemical systems, but appear to impact predominately GABAergic and glutamatergic transmission [1]. CB2 receptors are primarily found on immune cells in the periphery, and to some degree on certain cell types in the brain, largely microglia, but possibly neurons as well [2]. In addition, some eCB ligands are active at other receptor targets including peroxisome proliferator-activated receptor (PPAR) and type 1 vanilloid receptor (TRPV1), and can also directly affect the activity of some ion channels. Anandamide (AEA) and 2-arachidonoylglycerol (2-AG) are the two most well-studied eCB ligands and the most abundant eCBs found in the brain to date. AEA and 2-AG are synthesized and degraded by distinct enzymatic pathways (Figure 1).

In neurons, both AEA and 2-AG can be synthesized in an activity-dependent manner (Figure 1). Current conceptualizations of synaptic eCB signaling posit that eCBs are released from postsynaptic neurons during times of increased neuronal activity and serve to decrease afferent neurotransmitter release in a retrograde manner via activation of presynaptic CB1 receptors [3] (Figure 1). eCB synthetic enzymes and CB1 receptors are expressed in most limbic structures including the prefrontal cortex (PFC), amygdala, hippocampus, and nucleus accumbens. Moreover, synaptic retrograde signaling by eCBs has been described at glutamatergic and GABAergic synapses in each of these regions. Thus, eCBs are ideally situated to modulate synaptic integration and efficacy within limbic neurocircuitry, and thus exert profound effects on emotional and motivated behaviors.

The signaling life of eCB molecules is maintained by distinct hydrolytic enzymes, fatty-acid amide hydrolase (FAAH) and monoacylglycerol lipase (MAGL), which degrade AEA and 2-AG, respectively (Figure 1). There is also a growing belief that AEA and 2-AG may subserve distinct roles in the regulation of synaptic transmission, with AEA possessing a gatekeeper like 'tonic’ role whereby it regulates basal transmission and prevents excess transmitter release, while 2-AG represents the 'phasic’ signal that is brought online during periods of heightened neuronal activation and mediates most forms of excitation-induced eCB plasticity [4,5].

Genetic and pharmacological tools have been developed for the selective study of the role of CB1 receptors in the regulation of emotional behavior. In addition, pharmacological and genetic tools to modulate FAAH and MAGL to dissect the differential biobehavioral effects of AEA and 2-AG have become increasingly important in elucidating the role for eCBs in emotional and motivated behaviors [6]. Moreover, genetic studies and neuroimaging have begun to advance our knowledge of the key role eCBs play in the regulation of emotional responsivity in humans, and in the development of affective pathology. Here we will review preclinical and clinical data supporting a role for eCB lipids in the regulation of stress-response physiology, anxiety-like and depressive-like behaviors, and the adaptations observed in this signaling system in affective disorders in humans. Key open questions and future directions for preclinical and clinical research will be presented.

### Role of endogenous cannabinoids in emotionality and stress: preclinical studies

#### Anxiety-like behaviors

The general consensus of an abundance of preclinical studies is that eCB signaling constrains anxiety. CB1-/- mice appear more anxious in the standard behavioral measures of anxiety including the elevated plus-maze and light–dark box relative to wild-type mice under aversive testing conditions [7,8]; while under basal conditions CB1 deletion causes either an anxiogenic effect [9-13] or no effect [9,14,15]. Moreover, CB1-/- mice are resistant to the anxiolytic effects of benzodiazepines [11]. Further genetic dissection of the roles of CB1 receptors on specific neuronal subtypes indicate that deletion of the CB1 receptor from glutamatergic neurons does not affect anxiety in the elevated plus-maze, but does cause a slight anxiety-like phenotype in the open field under high environmental aversiveness [9]. However, genetic deletion of CB1 in glutamatergic neurons causes more anxiety upon repeated exposure to the open field under high light conditions, suggesting a role for CB1 on glutamatergic neurons in the habituation of anxiety behaviors to repeated exposure to aversive environments [9]. In contrast, mice lacking CB1 receptors on GABAergic neurons exhibit no differences in anxiety-like behaviors in the elevated plus-maze [16]. However, conditional mutant mice have revealed that CB1 receptors located on glutamatergic neurons are critical for the anxiolytic effects of low doses of exogenous CB1 receptor agonists, while CB1 receptors expressed by GABAergic neurons are critical for the anxiogenic effects of high doses of CB1 agonists [16]. Interestingly, targeted deletion of CB1 from serotonergic neurons alone seems to recapitulate the anxiety phenotype of global CB1-/- mice [17]. Overall, strong converging genetic studies indicate that CB1 receptors are important for reducing anxiety, particularly during times of high environmental aversiveness.

Pharmacological studies have for the most part mirrored the data obtained using genetic models. For example, acute administration of the CB1 receptor antagonists SR141716 or AM251 increase anxiety-like behavior in several behavioral measures, including the elevated plus-maze [18-22], novelty-induced hypophagia assay [23], social interaction test [24], light–dark box assay [25] and ultrasonic vocalization assay [26]. However, a few contradictory reports demonstrate anxiolytic actions of the CB1 receptor blockade. For example, a CB1 receptor antagonist reduced anxiety in the elevated plus-maze in maze-experienced mice [27], reduced some signs of anxiety in the defensive probe burying assay [28] and reduced anxiety associated with alcohol withdrawal [29]. However, all of these experiments involve an additional manipulation (such as cognitive training or alcohol withdrawal), which could explain the complex and contradictory effects of CB1 antagonism on anxiety therein.

The pharmacological blockade of eCB-degrading enzymes has been shown to exert anxiolytic actions in a variety of animal models. The first class of eCB degradation inhibitors targeted FAAH [30]. Blocking FAAH-mediated AEA degradation decreases anxiety in the elevated plus-maze [19,30-35], and the light–dark box test [36]; importantly, several studies indicate these effects are enhanced under conditions of high environmental aversiveness [34,37] or following exposure to stress [38,39]. FAAH inhibition also decreases anxiety in the rat pup ultrasonic vocalization test [26], light–dark exploration assay [25] and marble-burying assay [40]. Similarly, FAAH-/- mice exhibit reduced anxiety in the light–dark box assay and elevated plus-maze during conditions of high aversiveness, but not low stress conditions [31]. Unequivocally, the effects of pharmacological FAAH inhibition or genetic deletion are blocked by CB1 receptor antagonists [30,31], indicating that FAAH inhibition reduces anxiety through augmentation of AEA signaling at the CB1 receptor.

More recently, MAGL inhibitors that increase brain 2-AG levels have been evaluated in several models of anxiety. The MAGL inhibitor JZL-184 reduces anxiety in the marble-burying assay [40,41] and elevated plus-maze, under conditions of high environmental aversiveness [35,42-44], but not standard conditions [41]. Both CB1 and CB2 receptors have been implicated in the anxiolytic effects of JZL-184 [35,44]. Collectively, these data indicate that enhancement of eCB signaling, through either potentiation of AEA or 2-AG, reduces anxiety, particularly when brought on by stress or environmental aversiveness.

At a circuit level, the effects of eCB signaling on anxiety seem to be mediated by CB1 receptor signaling in the prefrontal cortex and amygdala, two structures commonly involved in the regulation of anxiety. Within the PFC, overexpression of FAAH reduces AEA levels and increases anxiety, while infusion of low doses of a FAAH inhibitor into the PFC reduces anxiety measures in the elevated plus-maze [45]. Similarly, administration of a CB1 receptor antagonist into the basolateral nucleus of the amygdala (BLA) increases anxiety [46], while inhibition of FAAH in the BLA reduces anxiety [47]. These data suggest that enhancement of eCB signaling within the PFC or amygdala can suppress anxiety, while disruption of eCB signaling in these structures can facilitate it. These findings are primarily based on the effects of manipulating AEA signaling; however, no studies to date have examined site-specific effects of MGL inhibition. As anxiety is typically associated with reduced activity in the PFC and enhanced activity in the amygdala, it is not surprising that electrophysiological evidence has demonstrated that eCB signaling in the PFC can enhance neuronal activity through a suppression of γ-aminobutyric acid (GABA) release [48,49], while suppressing glutamate release and excitability within the amygdala [50-53]. Accordingly, the current data would suggest that eCB signaling increases prefrontal cortical activity and suppresses amygdalar activation to dampen the expression of anxiety-like behaviors in rodents.

Overall, the totality of data regarding the role of eCB signaling in the modulation of anxiety behaviors suggest eCBs play particularly important roles in reducing anxiety under stressful or anxiogenic contexts, but also may contribute to constraining anxiety under non-stress conditions. In addition to general measures of anxiety, there is also a rapidly growing and very interesting literature regarding the role of the eCB system in fear learning and extinction; however, the complexity of this field is beyond the scope of the current review and there are several detailed reviews which focus exclusively on this topic (see [54] and [55]).

#### Depressive-like behaviors

Similar to the effects on anxiety behaviors, a role for eCB signaling in depressive-like behaviors has also been described. Augmentation of brain eCB signaling has been suggested as a novel antidepressant strategy by several authors [56-58]. In this section we will review the key studies implicating eCB signaling in depressive-like behaviors using acute models and tests – studies utilizing chronic stress will be discussed in the following section in the context of eCB modulation of stress responses.

CB1-/- mice have been shown to exhibit increases in passive coping responses in the forced swim test [59] and tail suspension test [60], although this is not a universal finding in the forced swim test at least [61,62]. Interestingly, CB1-/- mice have reduced hippocampal brain-derived neurotrophic factor (BDNF) [59,60], and normalization of hippocampal BDNF reverses the depressive-like phenotype of these mice in the tail suspension test [60]. These data, combined with data on the enhanced stress-sensitivity of these mice discussed below, suggest lifelong CB1 receptor deletion results in a depressive-like phenotype, and because of these data, it has been suggested that global CB1 deficient mice could represent a valid animal model for major depression [63,64]. However, more recent data indicate that selective deletion of CB1 from glutamatergic neurons actually has an antidepressant-like effect; whereas deletion of CB1 from CaMKIIα-expressing principal forebrain neurons or GABAergic neurons has no effect [65]. Interestingly, combined genetic and pharmacological studies have now revealed that the antidepressant effect of CB1 receptor deletion from cortical glutamatergic neurons is driven by an increase in serotonin release as it is reversed by serotonin depletion [66]. Clearly, the role of CB1 in the regulation of depressive-like behavior is complex, and CB1 receptors expressed by different subsets of neurons or neural circuits could even have opposing effects on depressive behavior.

Similar to genetic deletion, pharmacological inhibition of CB1 receptor activity has yielded complex and contradictory results. Some studies have demonstrated an antidepressant-like effect of the acute CB1 receptor blockade in the forced swim test and/or tail suspension test [67-69], while other reports indicate sub-chronic CB1 receptor antagonist treatments are required for consistent antidepressant-like effects [62]. In contrast, several studies have demonstrated no effect of CB1 receptor blockade in the forced swim test and tail suspension test [23,59,61,70-73], while one study demonstrated a depressive-like phenotype in the forced swim test after chronic CB1 antagonist treatment [74]. Similarly, some data support enhanced monoaminergic transmission after an acute CB1 receptor blockade [69], and global depletion of catecholamines can reverse the antidepressant effect of acute CB1 receptor antagonism [66]. Additionally, this effect of CB1 receptor antagonism is driven by CB1 receptors on GABAergic neurons as it is lost following selective deletion of CB1 from GABA neuronal populations [66], suggesting that the blockade of CB1 receptors on GABAergic neurons transiently increases catecholamine transmission to produce behavioral effects in the forced swim test. However, protracted administration of a CB1 antagonist, which produces a 'depressive’-like phenotype, actually decreased monoamine and BDNF levels [74], indicating that while acute effects may suggest an antidepressant response, clearly sustained inactivation of CB1 receptor signaling produces adverse changes in emotional behavior, such as increased anxiety, and evokes a biochemical signature similar to that seen following chronic stress.

Although a significant discrepancy exists between the antidepressant-like effects of the genetic and pharmacological CB1 receptor blockades, generally more consistent findings have been published with regard to the antidepressant-like effects of FAAH inhibition. Both acute pharmacological and lifelong genetic deletion of FAAH produce antidepressant-like effects in the forced swim test and tail suspension test [33,34,70,71,75,76]. Some studies have found that these effects are more robust under highly aversive testing conditions [34]. Interestingly, mice exposed to the forced swim test show rapid reductions in PFC AEA levels, and direct PFC injection of a FAAH inhibitor produced antidepressant-like effects in the forced swim test [76]. FAAH inhibition in the PFC increases the firing rate of serotonergic neurons, and pharmacological serotonin depletion prevents the antidepressant-like effects of FAAH inhibition in this assay [76,77]. Taken together these data suggest FAAH inhibition produces acute antidepressant-like effects via activation of serotonergic and noradrenergic signaling [58], and highlight the PFC again as a primary substrate through which these effects are mediated.

To date, no data are currently available on the antidepressant-like properties of MAGL inhibition, but these are likely currently underway. This is a critical question as it may reveal a second molecular target for the pharmacological targeting eCB signaling to give an antidepressant effect. Taken together, while the effects of CB1 receptor antagonism or deletion may be equivocal with respect to behavioral tests of stress coping, which is reflective of depressive behavior, there is a high degree of consistency among reports demonstrating that inhibition of FAAH produces antidepressant-like effects in rodent models.

#### Endocannabinoids and stress response modulation

The earliest studies into the role of eCB signaling in stress response physiology utilized CB1-/- mice, which exhibit a generally poorer coping response to stress. For example, CB1-/- mice exhibit greater reductions in sucrose intake during chronic unpredictable stress (CUS) than wild-type mice [13], and show enhanced behavioral inhibition and reduced ultrasonic vocalizations in response to repeated bell stress [78]. Similarly, chronic restraint stress-induced anxiety behaviors in the elevated plus-maze is augmented in CB1-/- mice, as is the stress-induced dendritic hypertrophy in the amygdala [12]. After chronic social defeat stress, mice lacking CB1 receptors on *single-minded-1*-expressing neurons of the basolateral amygdala and paraventricular hypothalamus show enhanced anxiety in the open field [79]. Furthermore, CB1-/- mice exhibit reduced foot-shock induced reinstatement of alcohol seeking behavior [80]. In contrast, other studies have found no effect of global CB1 deletion on anxiety behaviors after chronic social defeat stress [17]. Overall, these data suggest CB1 receptor deficiency impairs stress-coping responses and facilitates the development of stress-induced psychopathology.

Several studies have also investigated the effects of CB1 receptor antagonism on stress-induced behavioral dysregulation. For example, acute CB1 receptor blockade partially reverses the habituation of active coping responses observed during repeated restraint [81]. In addition, CB1 receptor antagonism exaggerates acute restraint stress-induced deficits in sucrose preference [82], with the magnitude of this effect intensifying with increasing duration of restraint stress exposure. In contrast, one study found an improved physical state and reduced anxiety in mice chronically treated with a CB1 receptor antagonist during CUS [68], while another found no effect of repeated CB1 receptor antagonist treatment on anxiety or hedonic measures after repeated social defeat stress [17].

In contrast to the above-mentioned behavioral studies, several authors have reported remarkably consistent effects of stress on brain regional eCB levels. The most consistent data indicate that acute and repeated restraint stress decreases AEA levels in multiple limbic brain regions but robustly in the amygdala [17,39,81,83-86]. In some cases more repeated bouts of stress produced larger decreases in AEA levels [81], and the reduction lasts at least 24 hours after termination of the last stressor [86]. Interestingly, the degree of stress-induced AEA reduction in the amygdala predicts stress-induced corticosterone elevation [85], suggesting a key role for amygdala AEA in the regulation of stress response physiology. The mechanisms regulating rapid reductions in AEA are not well understood; however, reductions in AEA after chronic stress are likely mediated via increased FAAH activity [39,84]. The effects of acute and repeated stress on 2-AG levels are also remarkably consistent between studies and laboratories. In most cases acute stress has little effect on 2-AG levels; however, repeated homotypic stress increases 2-AG levels in the amygdala and other limbic brain regions [17,84,86-88], while CUS has more variable effects [83], but often showing a decrease in hippocampal 2-AG levels [89]. Interestingly, chronic corticosterone treatment also increases amygdalar 2-AG levels [90]. These data suggest that, while AEA appears to be acutely sensitive to multiple forms of stress and responds with reduced tissue levels for possible protracted periods [85], 2-AG is increased after repeated homotypic stress exposure only, and this increase is transient and terminates after stressor discontinuation [86,88]. Furthermore, after repeated homotypic stress exposure, amygdalar 2-AG levels are inversely correlated with corticosterone secretion, and local antagonism of CB1 receptors within the basolateral amygdala reverses stress habituation, suggesting one function of the elevated 2-AG is to facilitate neuroendocrine habituation to repeated stress exposure [86].

Based on the totality of data reviewed this far, strong evidence points to eCB signaling as a stress-buffering system, and that one contribution to behavioral dysregulation induced by stress is an AEA-deficient state. Based on this simple interpretation several recent studies have examined the therapeutic potential of eCB modulation on stress-induced behavioral pathology. For example, repeated treatment of mice with the MAGL inhibitor JZL-184, which increases brain 2-AG levels, prevents repeated restraint stress-induced anxiety measured in the novelty-induced hypophagia assay [41]. Furthermore, both pharmacological and genetic inhibition of FAAH prevents chronic restraint stress-induced anxiety in the elevated plus-maze [39], as well as CUS-induced anhedonia [91]. Possible mechanisms of action of eCB augmentation in the mitigation of stress-induced behavioral dysregulation include modulation of hippocampal neurogenesis [92,93] and amygdala dendritic hypertrophy [39].

Taken together, this review of the preclinical research regarding the role of the eCB system in the regulation of stress and emotional behavior creates a compelling argument that eCB signaling acts to constrain activation of the stress response, and the ensuing neuroendocrine and behavioral responses to stress. Acute disruption of CB1 receptor signaling reliably increases anxiety and activation of the hypothalamic–pituitary–adrenal (HPA) axis, but has less consistent effects on depression-like behavior (which may be reflective of the behavioral tests being used), suggesting that there is an eCB tone acting to keep these processes suppressed in non-stressful environmental conditions. As AEA is believed to mediate the tonic actions of the eCB system, and because AEA levels are reduced in response to stress, our current theory is that AEA signaling acts as the 'gatekeeper’ of sorts, keeping stress and anxiety at bay, likely through its actions in the amygdala. In response to stress, particularly stress of high emotional load or repeated exposure to a common stressor, there is a mobilization of 2-AG, which seems to be important for restricting the magnitude of the stress response and aiding in normative recovery to pre-stress levels of functioning. Accordingly, impairments in eCB signaling, at either the ligand or the receptor level, would likely result in either the induction of an undue activation of stress responsive systems (such as the amygdala) under non-threatening conditions, or an impairment in appropriate stress adaptation, both of which could predispose an organism or individual to exaggerated effects of stress. As such, these data would suggest that eCB signaling could be compromised in stress-related affective illnesses (such as major depression or post-traumatic stress disorder, both of which appear to be driven by maladaptive responses to stressful life events), and that pharmacological enhancement of eCB signaling could be a novel therapeutic avenue for the treatment of these neuropsychiatric conditions. It should be noted, however, that the majority of the studies investigating the effects of increased eCB signaling largely focus on FAAH, and only a few recent studies have begun to examine the effects of MAGL inhibition. Given that there are noted pharmacokinetic, and possibly functional, differences between AEA and 2-AG [4-6], it should not be immediately assumed that inhibition of either enzyme will produce synonymous effects (although the initial studies do suggest a high degree of similarity). With the development of more specific tools to target FAAH and MAGL exclusively, how these two signaling molecules can be harnessed for the therapeutic treatment of mood and anxiety disorders will become clearer. The following section will discuss the translational and clinical evidence that has been gathered to date and which supports the data generated from preclinical studies and demonstrates the importance of the eCB system in the regulation of stress responses and emotional behavior in humans.

### Role of endogenous cannabinoids in emotionality and stress: clinical studies

In humans, centuries of cannabis use for the purposes of controlling stress and anxiety strongly suggest that activation of the human eCB system serves a similar function to constrain stress and anxiety circuits as has been demonstrated in animal models. In fact, the primary reason as to why most individuals consume cannabis on a regular basis is because of its ability to reduce feelings of tension, promote relaxation and take the edge off stressful life events [94]. More so, clinical studies employing direct CB1 receptor agonists have shown potential therapeutic benefit in the treatment of both generalized anxiety conditions and post-traumatic stress disorder (PTSD) [95,96]. Interestingly, neuroimaging studies have replicated the findings of preclinical studies, demonstrating that cannabinoid administration, or regular cannabis use, can effectively dampen activation of the amygdala in response to threatening or aversive stimuli [97-99]. Similarly, cerebral blood flow studies have shown that cannabinoids can increase activation of frontal cortical regions including the anterior cingulate [100], which agrees with the rodent studies demonstrating that CB1 receptor activation can augment prefrontal cortical activity [48,49]. As such, from a translational perspective these data support those generated from animal studies demonstrating that activation of the CB1 receptor in humans can reduce anxiety, deactivate the amygdala and enhance prefrontal cortical function. With respect to how the eCB system itself may function in these regards, there are three lines of evidence that can be drawn on to determine the putative role of this system in the endogenous regulation of stress and emotional behavior: 1) pharmacological challenge studies in which the CB1 receptor is blocked to unmask the role of eCB signaling, 2) biochemical studies investigating dynamic or steady-state changes in eCB ligand content in response to stress or in psychiatric conditions and 3) genetic studies investigating the effect and possible role of functional polymorphisms in the eCB system in response to stress or in psychiatric illnesses. Each of these three lines of evidence will be discussed below.

### Pharmacological studies of CB1 receptor blockade in humans

As with the studies performed in rodents, the effects of CB1 receptor antagonism in humans generally support the hypothesis that eCB signaling negatively regulates stress and anxiety. Clinical development of CB1 receptor antagonists was initially advanced as a putative mechanism to treat obesity, given that eCB signaling promotes feeding and weight gain, and that a blockade of eCB signaling in animals could mitigate the effects of diet-induced obesity. In humans, clinical studies have clearly demonstrated that blocking CB1 does provide some therapeutic benefit in promoting weight loss and alleviating metabolic abnormalities associated with obesity; however, the first CB1 receptor antagonist tested, rimonabant, developed specifically for this purpose, was ultimately removed from the market due to the development of anxiety and depressive symptoms in a significant proportion of individuals [101,102]. In fact, a meta-analysis of the four major clinical studies performed with rimonabant found that there was approximately a threefold increase in the emergence of anxiety symptoms in patients receiving rimonabant versus a placebo, and these studies were all performed on individuals who had no history of psychiatric illness [103]. The largest multi-center trial for rimonabant (involving over 18,000 patients in 42 countries) similarly found that there was a significant increase in neuropsychiatric side effects (in approximately one-third of patients treated with rimonabant) and serious psychiatric side effects (which developed in roughly 1 in 40 individuals treated with rimonabant) following CB1 receptor antagonism [104]. One case report even discusses the *de novo* emergence of a profound bout of melancholic depression, which occurred following administration of rimonabant, and subsided following cessation of drug administration [105]. Taken together, these data clearly demonstrate that disruption of eCB signaling in humans is capable of increasing the signs of anxiety and depression, which supports the hypothesis that eCB signaling in humans, as in rodents, acts to dampen negative emotions.

Given the psychiatric disturbances found in the obesity trials, it became quite difficult to ethically undertake an in-depth study of the effects of CB1 receptor antagonism in humans on facets of stress and emotional behavior. However, a few studies have emerged and they have shed some insight into the possible mechanisms. First, one report found that high doses of rimonabant were capable of increasing cortisol in some subjects [106], supporting the animal studies indicating that eCB signaling negatively regulates activation of the HPA axis [87]. Second, a series of studies combining imaging and cognitive testing demonstrated several interesting effects of CB1 receptor antagonism in humans, which could relate to its ability to promote depression. Specifically, a 7-day treatment regimen with a CB1 receptor antagonist was found to blunt activation of reward circuits in the brain in response to pleasurable stimuli [107], suggesting that deficient eCB signaling could be a putative mechanism for anhedonia in depression. Similarly, both a single dose of rimonabant, as well as a 7-day treatment regimen of rimonabant, were not found to affect mood significantly, *per se*, but they were found to suppress the recall of emotionally positive memories [108] and promote negative bias in memory recall [109]. Both effects could result in a negative emotional bias, a phenomenon commonly seen in affective illnesses and known to be a risk factor for the development of major depression.

Accordingly, these pharmacological studies in humans demonstrate that disruption of eCB signaling is sufficient to promote anxiety, increase HPA axis activity, impair reward processes in the brain and enhance negative emotional memory biases, all of which are all hallmark signs of mood disorders. Thus, consistent with data obtained from animal studies, these data support the possibility that impaired eCB activity could be a predisposing factor for the development of stress-related neuropsychiatric conditions.

### Biochemical studies of the endogenous cannabinoid system in mood and anxiety disorders

Another way to examine the eCB system in humans is to measure eCB ligand content (AEA and 2-AG) in the circulation of individuals to see how it is affected by stress or altered in psychiatric disorders. Again, as was seen in the pharmacological studies in humans, the data generated to date are largely consistent with what has been found in preclinical studies. First, several studies have demonstrated that circulating levels of eCB molecules are responsive to stress. Hill and colleagues [110] demonstrated that exposure to the Trier social stress test resulted in a significant increase in 2-AG concentrations (and a small trend toward increased AEA concentrations) in the circulation. Another study used parabolic flight as a physiological stressor and found that it increased 2-AG in the circulation, and that individuals who did not mount a 2-AG response to the stress exposure exhibited dramatically higher levels of cortisol, suggesting that impairments in the natural induction of an eCB response to stress produce heightened physiological stress responses [111]. A more recent report that also examined eCB responses in the circulation to the Trier social stress test, demonstrated that circulating levels of AEA, but not 2-AG, increased in response to stress [112]. Interestingly, as in the parabolic flight study, this study also reported that lower basal levels of AEA predicted increased HPA responses to stress, again suggesting that eCB signaling negatively relates to the magnitude of the stress response [112]. As such, these studies demonstrate that eCB signaling in humans is responsive to stress, and that failure of this system to be appropriately engaged results in increased responses to stress.

With respect to direct associations of eCB levels with mood and anxiety disorders, several studies do generally seem to suggest that there is a frank deficiency in systemic eCB signaling in several psychiatric conditions. Two independent reports have found that circulating levels of 2-AG (and in one study also AEA) are reduced in medication-free women who have been diagnosed with major depression [110,113]. Interestingly, circulating levels of AEA and 2-AG were both found to be elevated in individuals with minor depression [113], which suggests the hypothesis that active recruitment of the eCB system may curb the development of frank major depression. Like these reports, it has also been found that in individuals who have undergone cardiac surgery, the subsequent development of depression following surgery was related to low levels of circulating AEA and 2-AG during the perioperative period [114].

Less research has directly examined eCB levels in anxiety conditions. Interestingly, two studies have both reported that basal levels of AEA in the circulation negatively correlate with anxiety scores on clinical scales, both in a healthy population [112] and in one composed of individuals with major depression [113]. That is, individuals with higher levels of anxiety have lower levels of circulating AEA. This is consistent with the preclinical studies that have found relations between stress and anxiety with low levels of AEA [39,45,81,85]. More interestingly, a recent report found that circulating levels of AEA are significantly reduced in individuals with PTSD, compared to both healthy controls and those exposed to trauma who did not develop PTSD [115]. This reduction in circulating AEA was also significantly correlated with upregulation of CB1 receptors throughout limbic circuits in the brain [115], suggesting that: (1) circulating levels of eCB may be reflective of centrally active eCBs and (2) deficient eCB signaling may result in a compensatory upregulation of CB1 receptors in the brain. Consistent with this, we have recently demonstrated that PTSD is associated with reduced levels of 2-AG in the circulation, while lower AEA levels correlated with the intensity of intrusive symptoms [116]. One recent report, however, found the opposite effect with increased levels of AEA and 2-AG in individuals with PTSD [117]; it is not clear what the discrepancy is between this study and the previous two, but could relate to differences in disease severity, as was seen in the minor versus major depression study [113].

In sum, the majority of studies examining eCB levels in humans have come to two conclusions that are largely consistent with the preclinical literature. First, eCB signaling is responsive to stress and deficient eCB activity is associated with increased stress responses, suggesting that eCB signaling constrains the magnitude of the stress response. Second, basal eCB signaling appears to be reduced in individuals afflicted with stress-related psychiatric conditions, such as major depression or PTSD, suggesting that impaired eCB signaling is related to the development of these conditions. Whether the reduction in eCB signaling is a predisposing factor or a burden of the disease itself, is yet to be determined, as is whether these changes in circulating eCB levels are reflective of central eCB activity. The overall uniformity of these findings supports the preclinical studies and suggests that a functional eCB system is required for appropriate adaptation and buffering of stress in humans.

### Genetic studies of the endogenous cannabinoid signaling in stress-responsive systems and stress-related disorders

An additional source of information that provides insight into the role of eCB signaling in humans with respect to stress and stress-related psychiatric illnesses is genetic studies. With respect to the CB1 receptor (*CNR1* gene), several studies to date have examined different polymorphisms in the *CNR1* gene and how they relate to psychiatric illness, particularly depression. One polymorphism in particular, rs1049353, has received a fair amount of attention. This allele has a major form (G allele) and a minor form (A allele), and it would appear that the A allele of this polymorphism exerts some level of protection against stress and depression. Specifically, one report has demonstrated in two separate populations that carriers of the A allele are protected against the development of anhedonia and major depression in adulthood following early life stress or abuse [118]. It should be noted, however, that this effect was not entirely replicated by a second group, although they did note that was a moderate risk reduction in carriers of the A allele, but they suggested that this allele may be more specific for anhedonia than depression, *per se*[119]. Consistent with this idea that the minor allele of this polymorphism is protective, Domschke and colleagues (2008) demonstrated that individuals carrying the G allele were more likely to exhibit antidepressant resistance than those with the A allele [120], suggesting that the A allele may confer greater antidepressant responsiveness. This effect was found primarily in females, and especially those that presented with melancholic depression with high anxiety [120]. Interestingly, a replication study from another group found the opposite effect: that if you were male and a G allele carrier you were more likely to exhibit a better antidepressant response than if you were an A allele carrier, suggesting that there may be some sexual divergence in the role of the eCB system in depression and antidepressant treatment [121]. Of note, while not fully explored because the effect did not quite achieve statistical significance, another report also found that presence of the A allele in this polymorphism reduced the development of depression in association with exposure to stressful life events [122]. More interestingly, carriers of the G allele were found to exhibit blunted limbic and striatal activation to happy faces in imaging studies [123], which is a common feature in depression and is associated with blunted reward responsiveness and anhedonia. Taken together, these studies would suggest that the A allele in the rs1049353 polymorphism of the *CNR1* gene results in some level of protection against the development of depression, particularly in response to stress exposure.

The one issue surrounding the rs1049353 polymorphism is that there is no knowledge regarding whether it is functional or not. This polymorphism is exonic, but it is a synonymous polymorphism (in that the base pair substitution does not result in a difference in protein sequence), so it is unclear what effects it has, if any, on CB1 receptor densities *in vivo*; however, it has been postulated that it may affect mRNA stability [120]. Based on the preclinical literature and the general consensus that eCB signaling confers protection against the effects of stress, one possible interpretation is that the A allele of this polymorphism could result in more stable mRNA and a subsequent increase in protein expression and function of the CB1 receptor *in vivo*; however, this remains to be experimentally demonstrated.

Consistent with this hypothesis, other polymorphisms in the *CNR1* gene have been shown to have functional effects on receptor expression. For example, carriers of the C allele of another polymorphism in the *CNR1* gene (rs2023239) have been found to exhibit increased CB1 receptor binding both in post mortem brain tissue [124] and *in vivo* through positron emission tomography imaging studies [125]. Interestingly, carriers of the C allele (which have greater CB1 receptor density) have also been found to have increased hippocampal volume [126]. As hippocampal volume is known to be reduced in major depression [127], and reduced hippocampal volume is thought to be a risk factor in PTSD [128], these data would suggest that polymorphisms in the *CNR1* gene that result in greater CB1 receptor activity may result in increased hippocampal volume and thus provide a neural substrate mediating a protective effect. CB1 receptor activation is known to drive neurogenesis in the hippocampus [129] and also protect hippocampal neurons from excitotoxic damage [130], and so it is reasonable to predict that greater CB1 receptor densities could result in enhanced hippocampal function. Unfortunately, as there are no studies to date examining the effect of the rs2023239 polymorphism on psychiatric illness it is not known if this polymorphism has any effect on disease vulnerability. Future studies should thoroughly investigate the role of this polymorphism in psychiatric illness, and also determine if the rs1049353 polymorphism has a similar effect on CB1 receptor density in humans to understand how these gene variants could impact disease progression.

In addition to this abundance of work on the rs1049353 polymorphism, a scattering of other polymorphisms of the *CNR1* gene have been investigated. For examples, the T allele in the rs7766029 polymorphism is significantly associated with the development of depression following increasing exposure to stressful life events [122]. With respect to anxiety, the only study that has been done with the *CNR1* gene is one that identified an epistatic relation between *CNR1* and the serotonin transporter. Specifically, they found that individuals who possessed the short variant of the serotonin transporter, coupled to the GG homozygous allele of the rs2180619 polymorphism of the *CNR1* gene, exhibited dramatically higher levels of anxiety [131]. They suggested that this GG allele resulted in lower levels of CB1 receptor expression, which could result in excess serotonin release, and when coupled with the short serotonin transporter allele (which exhibits impaired 5-HT clearance from the synapse), could result in excessive serotonin signaling that would produce heightened levels of anxiety [131]. It is of interest to note that this model is consistent with the recent findings that CB1 receptor deletion exclusively from serotonergic neurons was sufficient to produce a state of anxiety [17], suggesting that interactions between eCB signaling and serotonin are indeed important for the regulation of anxiety. While very intriguing, all of these polymorphisms require more research to validate that these effects are replicable and more importantly, what the functional effects of all these polymorphisms are. Regardless, these data do indicate that genetic variation in the CB1 receptor does relate to changes in emotional behavior and thus further supports the evidence that eCB signaling is an important contributor of emotional behavior in humans.

One of the most interesting gene variants in the eCB system studied to date is a functional polymorphism in the *FAAH* gene. The C385A polymorphism of *FAAH* is when an A base is substituted for a C base in the sequence, resulting in a non-synonymous change of proline for threonine in the FAAH protein [132]. The A allele results in enhanced proteolytic degradation of FAAH [132], which results in lower levels of the FAAH protein and activity [133] and higher levels of AEA in the circulation [134]. Interestingly, carriers of the A allele of this *FAAH* polymorphism exhibit blunted activation of the amygdala in response to threat cues and enhanced activation of the ventral striatum in response to reward-related cues [135]. That is, stress-induced activation of the amygdala is reduced in those who have elevated AEA levels, and striatal responses to reward are higher. Subsequent research on this polymorphism has also revealed that A carriers exhibit lower levels of trait anxiety, reduced stress reactivity and also demonstrate more rapid habituation of amygdalar activation in response to aversive cues than C carriers [136]. However, one recent report found the exact opposite effect and showed increased startle reaction in A carriers to unpleasant images, suggestive of greater amygdala activation; however, the authors did not measure amygdala activation through neuroimaging techniques and based their assumptions purely on behavioral outcomes [137]. The reason for this putative discrepancy, and possible interactions with life stress, should be considered in future studies. The findings that high levels of AEA reduce amygdala reactivity to stress, reduce trait anxiety and promote habituation to stress are highly consistent with the preclinical studies detailed above.

## Conclusions

Ultimately, these studies on eCB signaling in humans generally agree with the preclinical findings and suggest that in humans, eCB signaling is important for regulating stress and emotions. Elevated eCB signaling seems to be associated with reduced stress and anxiety, while impaired eCB signaling is associated with greater vulnerability to stress, anxiety and depression. The extent to which these findings can be used on a translational platform to understand the pathophysiology of stress-related psychiatric conditions in humans has yet to be determined, but the convergence of the preclinical and clinical findings detailed here strongly suggests that this should be a field of focused research in coming years.

We propose several areas of research that would help to fill gaps in knowledge and guide research to further determine the therapeutic utility of the eCB system with respect to the development of novel therapeutics for mood and anxiety disorders. First, what are the relative benefits and drawbacks of selective AEA and 2-AG augmentation, and would dual inhibition of FAAH and MAGL be a more efficacious approach? Second, what are the downstream signaling mechanisms responsible for the anxiolytic, antidepressant and anti-stress effects of FAAH and MAGL inhibition? Third, what is the precise molecular mechanism subserving the context-dependency of eCB modulation of anxiety behaviors? We and others have suggested a key factor is the reduction in AEA signaling that occurs under stressful conditions [138]; however, the mechanisms subserving these effects are not well understood. Fourth, are eCB augmenting agents able to prevent, and more importantly, reverse, stress-induced pathology? If effective, do they extend beyond behavioral effects to metabolic, cardiac and immune effects of chronic stress? Is there a causal relationship between stress-induced adaptations in eCB levels and stress-induced pathology? Finally, are there other molecular targets that can be utilized to enhance eCB signaling for therapeutic gain? With regard to this last question, very recent studies suggest that, in addition to FAAH and MAGL, COX-2 could also be a viable target for eCB augmentation and have anxiolytic potential [136]. Taken together, there is a very compelling argument forming that eCB signaling is a vital component of stress-regulatory systems in mammals, including rodents and humans. As such, the eCB system represents an ideal system for translational research since human studies generally corroborate animal studies. Animal studies can delve further into the mechanism and inform future clinical studies investigating the role of eCB signaling in treating psychiatric disorders. Only time, and clinical trials, will tell if this system truly represents a novel therapeutic target for mood and anxiety disorders.

## Abbreviations

2-AG: 2-arachidonoylglycerol; AEA: Anandamide; BDNF: Brain-derived neurotrophic factor; BLA: Basolateral nucleus of the amygdala; CB1: Cannabinoid type 1; CB2: Cannabinoid type 2; CUS: Chronic unpredictable stress; eCB: Endogenous cannabinoid, FAAH, fatty-acid amide hydrolase; GABA: γ-aminobutyric acid; HPA: Hypothalamic–pituitary–adrenal; MAGL: Monoacylglycerol lipase; PPAR: Peroxisome proliferator-activated receptor; PFC: Prefrontal cortex; PTSD: Post-traumatic stress disorder; TRPV1: Type 1 vanilloid receptor.

## Competing interests

The authors declare that they have no competing interests.

## Authors’ contributions

Both authors contributed and edited the entire manuscript, but SP primarily wrote the preclinical studies section and MNH primarily wrote the clinical studies section. Both authors read and approved the final manuscript.
